# PyHFO 2.0: an open-source platform for deep learning—based clinical high-frequency oscillations analysis

**DOI:** 10.1088/1741-2552/ae10e0

**Published:** 2025-10-27

**Authors:** Yuanyi Ding, Yipeng Zhang, Chenda Duan, Atsuro Daida, Yun Zhang, Sotaro Kanai, Mingjian Lu, Shaun Hussain, Richard J Staba, Hiroki Nariai, Vwani Roychowdhury

**Affiliations:** 1 Department of Electrical and Computer Engineering, University of California, Los Angeles, CA, United States of America; 2 Division of Pediatric Neurology, Department of Pediatrics, UCLA Mattel Children’s Hospital, David Geffen School of Medicine, Los Angeles, CA, United States of America; 3 Department of Neurology, UCLA Medical Center, David Geffen School of Medicine, Los Angeles, CA, United States of America; 4 Department of Civil Engineering, University of California, Los Angeles, CA, United States of America

**Keywords:** neurophysiology, EEG, high-frequency oscillation, deep learning, open-source software, graphical user interface

## Abstract

*Objective.* Accurate detection and classification of high-frequency oscillations (HFOs) in electroencephalography (EEG) recordings have become increasingly important for identifying epileptogenic zones in patients with drug-resistant epilepsy. However, few open-source platforms offer both state-of-the-art computational methods and user-friendly interfaces to support practical clinical use. *Approach.* We present PyHFO 2.0, an enhanced open-source, Python-based platform that extends previous work by incorporating a more comprehensive set of detection methods and deep learning (DL) tools for HFO analysis. The platform now supports three commonly used detectors: short-term energy, Montreal Neurological Institute, and a newly integrated Hilbert transform-based detector. For HFO classification, PyHFO 2.0 includes DL models for artifact rejection, spike HFO detection, and identification of epileptogenic HFOs. These models are integrated with the Hugging Face ecosystem for automatic loading and can be replaced with custom-trained alternatives. An interactive annotation module enables clinicians and researchers to inspect, verify, and reclassify events. *Main results.* All detection and classification modules were evaluated using clinical EEG datasets, supporting the applicability of the platform in both research and translational settings. Validation across multiple datasets demonstrated close alignment with expert-labeled annotations and standard tools such as RIPPLELAB. *Significance.* PyHFO 2.0 aims to simplify the use of computational neuroscience tools in both research and clinical environments by combining methodological rigor with a user-friendly graphical interface. Its scalable architecture and model integration capabilities support a range of applications in biomarker discovery, epilepsy diagnostics, and clinical decision support, bridging advanced computation and practical usability.

## 1. Introduction

High-frequency oscillations (HFO) in intracranial electroencephalography (EEG) have been increasingly recognized as a promising biomarker for the localization of epileptogenic zones in human and animal studies [1–5]. A growing body of evidence suggests that the resecting of HFO-generating regions correlates with improved postoperative seizure outcomes, highlighting the clinical relevance of accurate HFO detection and classification [6–17]. Beyond surgical planning, HFOs have been explored as broader biomarkers of brain network dynamics [18] and treatment response [19]. At the same time, recent reviews emphasize that important challenges remain in validating HFOs and related biomarkers for widespread clinical use [20]. In this context, automatic HFO detection is still prone to false positives, and distinguishing clinically relevant events from noise or physiological activity often requires both computational model assistance and interactive visualization tools to support expert review.

During the past decade, multiple software tools have been developed to assist in HFO detection, visualization, and analysis. Among them, RIPPLELAB [21] stands out as an influential open-source MATLAB-based application that consolidated the mainstream HFO detection algorithms along with basic EEG visualization features. The accessibility of RIPPLELAB allowed many studies to standardize HFO detection methods [22–27], accelerating the adoption of HFO research in various clinical and research settings. Concurrently, multiple open-source EEG processing frameworks -such as MNE [28], YASA [29], and PyEEG [30]—emerged to support various neurophysiological biomarkers. However, despite recent advances in DL for HFO classification, existing open-source frameworks lag behind in integrating these techniques into practical applications.

To bridge the gap between these evolving methods and clinical utility, our previous work introduced a software platform **PyHFO** [31] as a foundational step towards integrating epilepsy research, particularly HFO studies, with advanced signal processing and DL techniques. However, the initial version had several limitations. It lacked a user-friendly interface and did not support integration between model predictions and expert review or annotation. While the platform offered a scalable architecture, it provided only a limited selection of detectors and classifiers. A truly effective system requires the ability to incorporate new detection methods, classification models, or biomarkers to reflect the heterogeneous and evolving landscape of HFO research. Ideally, such a platform should also integrate seamlessly with modern machine learning ecosystems, enabling researchers to deploy state-of-the-art tools with minimal technical overhead.

In this paper, we present **PyHFO 2.0**, a significantly upgraded version of our original platform to further improve clinical engagement by adding: (1) an annotation window that enables in-depth review, verification, and manual annotation of detected HFO events; (2) a pipeline enabling users to train or fine-tune their own DL classifiers, facilitate user-driven model customization, incorporating models hosted on Hugging Face for automatic downloading and inference; (3) demonstration of such pipeline by incorporating a state-of-the-art epileptogenic HFO (eHFO) classifier into the ecosystem; (4) further enhance functionalities of PyHFO by adding a new Hilbert-based detector and adding user interface (UI) designed for greater intuitiveness and expanded scalability. These new features underscore the growing emphasis on flexible, user-friendly software that can adapt rapidly to both clinical and research demands. By integrating with the Hugging Face ecosystem, PyHFO 2.0 also establishes a direct link to the broader machine learning community, encouraging faster adoption of cutting-edge models for EEG analysis. In summary, PyHFO 2.0 differs fundamentally from both the original PyHFO and existing tools by unifying multiple detection methods, DL–based classification with Hugging Face support, and an interactive annotation interface. Together with a streamlined graphical UI (GUI), these improvements make PyHFO 2.0 the first open-source platform to combine DL with practical clinical usability for HFO analysis.

The remaining sections of this paper are organized as follows. First, we outline the overall workflow of PyHFO 2.0. We then detail each supported functionality. Next, we present the validation and benchmarking results for these methods, demonstrating their performance across diverse datasets. Finally, we discuss potential extensions–such as cursor support for signal inspection, broader biomarker integration, and the adoption of newer DL techniques–and conclude by considering how PyHFO 2.0 can further accelerate research and clinical translation in epilepsy care. To begin, we describe the datasets and methods used to evaluate the platform.

## 2. Methods and materials

### 2.1. Data acquisition

EEG data used for software development, model training, and evaluation were drawn from three datasets previously described in our earlier work [31].

**UCLA iEEG Dataset:** Intracranial EEG recordings from 19 patients (10 female, 9 male; ages 3–20 years) with drug-resistant focal epilepsy, each consisting of a 10 min segment during slow-wave sleep. Data were acquired using grid/strip electrodes at a 2000 Hz sampling rate [12, 32].

**Zurich iEEG HFO Dataset:** Five-minute interictal sleep EEG segments from 20 patients (16 female, 14 male; age range: 17–52 years), recorded with both grid/strip and depth electrodes, originally sampled at 4000 Hz and downsampled to 2000 Hz. Bipolar referencing followed the procedure in [33].

**UCLA Rodent Dataset:** Intracranial EEG (iEEG) recordings were obtained from two adult male Sprague–Dawley rats (300–350 g) using depth electrodes targeting the neocortex and hippocampus. One animal received a traumatic brain injury, while the other served as a sham control. [34].

### 2.2. Requirements

The PyHFO 2.0 software is a multi-window GUI developed in PyQt. It is intended to be a user-friendly and intuitive tool that users with technical and non-technical backgrounds can use to detect and classify HFOs in a time-efficient way. PyHFO 2.0 has been released under Academic Licenses (Licenses for Sharing Software Code Non-commercially, UCLA TDG). The GUI interface was implemented in PyQt version 5.15 to make it compatible with hardware platforms such as Mac OS, Linux, and Windows. The HFO detectors were implemented in Python 3.9.0, and the DL-based classifier was implemented in PyTorch 2.0 and Transformers 4.49.0. We chose Python as the programming platform because it is widely used in large-scale data analysis and DL in the medical image field. PyHFO 2.0 currently supports EEG data in EDF and BrainVision file formats, which are among the most widely used in clinical and research EEG. Other formats (e.g. BDF, CNT) are not yet directly supported and require prior conversion.

### 2.3. Code availability

The open-source software package is freely available on GitHub (https://github.com/roychowdhuryresearch/pyHFO/tree/pyBrain), and the deep learning (DL) model checkpoints used for classification are hosted on Hugging Face (https://huggingface.co/roychowdhuryresearch).

### 2.4. Getting started

To help users become familiar with PyHFO 2.0, we provide a set of instructional videos that walk through the graphical interface, highlight key features, and demonstrate how to process and analyze EEG data step by step. For users interested in customizing the DL component, two example scripts are included. One script shows how to train a new classification model using the platform’s built-in data format, while the other provides a way to integrate an existing pre-trained model into the software without requiring additional training. These resources are included in the GitHub repository (see Code Availability).

### 2.5. Overview

**Figure 1.**
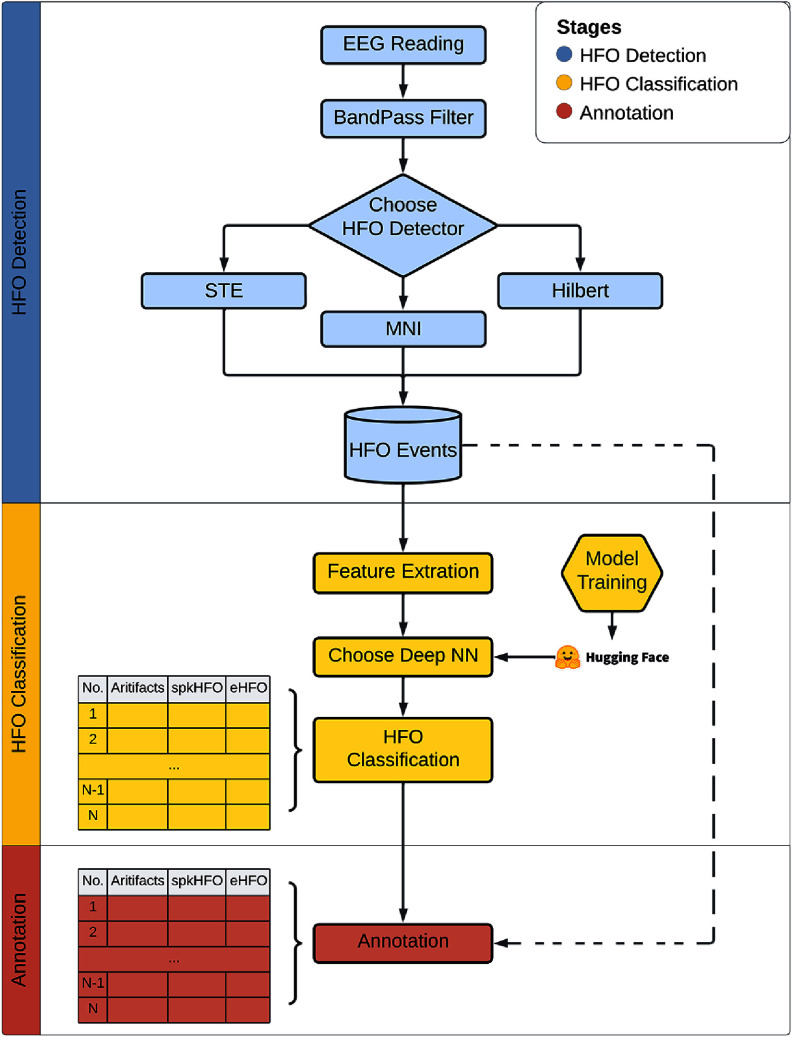
Overview of the workflow. This flowchart illustrates the three primary stages of our study: HFO detection, deep learning classification, and annotation. The workflow significantly streamlines the efficiency of HFO analysis. Specifically, HFO detectors identify HFO events from EEG recordings and return the start and end timestamps of the detected events. The EEG signal of each detected events are sent to pre-trained deep learning models hosted on the Hugging Face Hub for HFO classification. Finally, the results can be reviewed and modified using the annotation functionality.

The complete workflow is illustrated in figure 1. Upon loading an EEG recording, PyHFO 2.0 operates through three primary stages: HFO detection, DL-based HFO classification, and results annotation. The details of each critical stage are elaborated in the following sections. To facilitate practical adoption, we include detailed descriptions of the interface and workflows, so that the paper can also serve as a standalone reference for end-users.

## 3. Results

### 3.1. New features in PyHFO 2.0

PyHFO 2.0 builds on the existing PyHFO framework with three major enhancements. First, it integrates the Hilbert [35] method, a widely adopted HFO detection technique, thereby expanding the platform’s range of detection algorithms. Second, it introduces a new DL model specifically designed to detect epileptogenic HFOs (eHFOs) [12] with greater accuracy, and integrates all three classification models (artifact, spike HFO (spkHFO), and eHFO) into the Hugging Face ecosystem for automated download and streamlined model management. Third, an annotation feature has been implemented that allows users to interactively review, verify, and refine detection and classification results. A detailed verification of the newly added Hilbert detector and the eHFO classification model is provided in Clinical Validation. In the sections that follow, we offer a comprehensive guide to using PyHFO 2.0. Beyond covering these newly added features, we detail all core functionalities of the platform, equipping users with the knowledge and practical steps needed to make full use of its capabilities.

### 3.2. EEG loading and display

**Figure 2.**
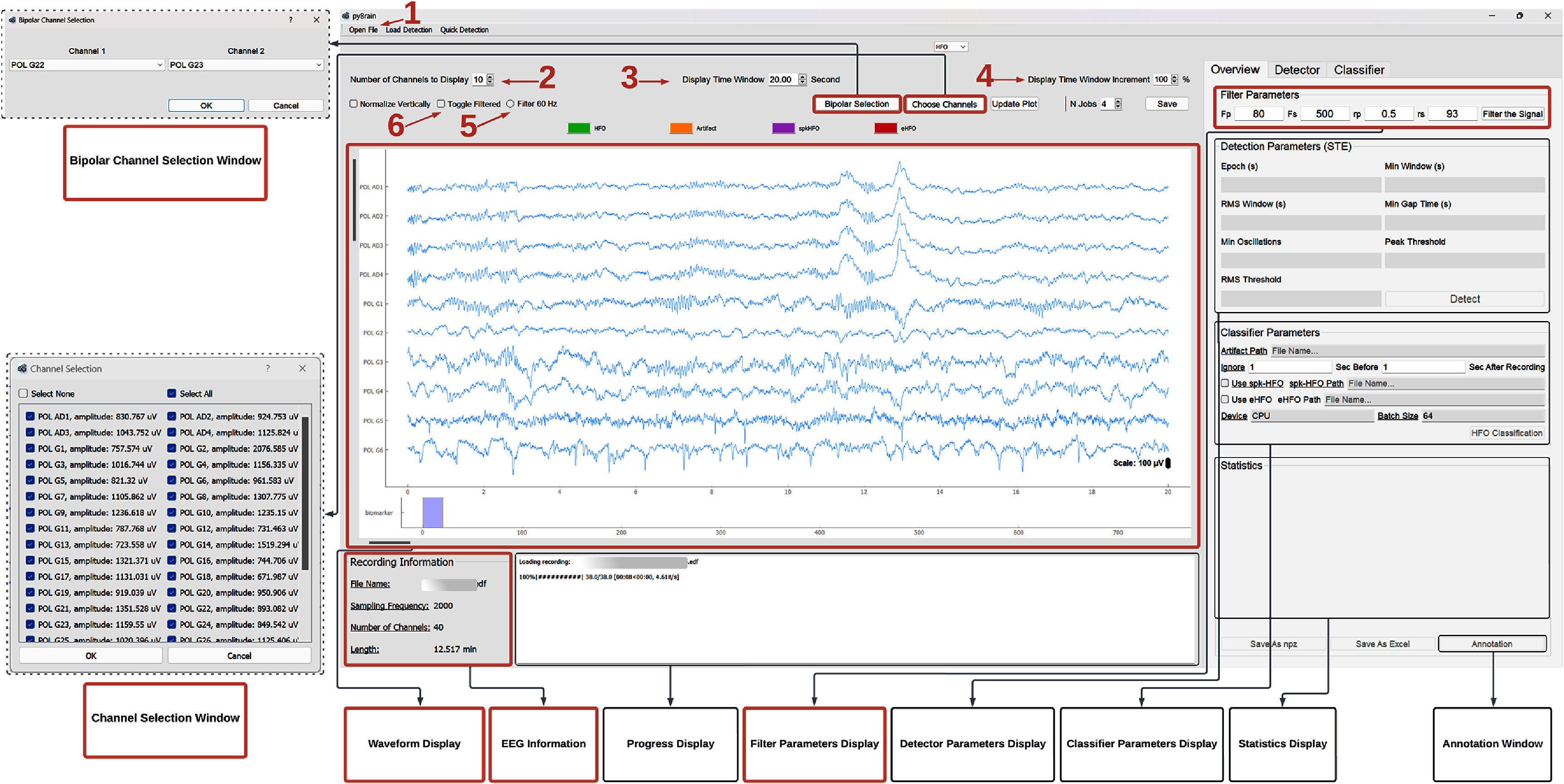
EEG loading and display EEG loading and display functionalities are highlighting in red boxes. Arrow 1 indicates the ‘open file’ button, which loads an EDF file into the ‘waveform display’ panel. The ‘Recording Information’ panel displays basic information about the loaded file. A vertical scroll bar lets users navigate across channels, and a horizontal scroll bar supports time-based exploration of the EEG data. Arrows 2 and 3 mark the fields for selecting how many channels and how much time are displayed, while arrow 4 adjusts the time scroll increment. The ‘Choose Channels’ button opens a window for selecting specific channels to view, and the ‘Bipolar Selection’ button opens a window allowing users to form bipolar-referenced signals between any two channels. In the ‘Filter Parameters’ panel, users can configure bandpass filter parameters. Separately, the 60 Hz interference option (arrow 5) allows removal of power-line noise. Finally, arrow 6 points to the ‘Toggle Filtered’ checkbox, enabling users to switch between raw and filtered signals for comparison.

PyHFO 2.0 supports loading multi-channel EEG data in EDF formats and provides an interactive waveform display with recording metadata such as file name, sampling frequency, number of channels, and recording length. Users can adjust channels, apply bipolar referencing, configure filters, and navigate recordings efficiently. These visualization features support rapid inspection of raw and preprocessed signals and provide a foundation for subsequent detection and classification. A detailed breakdown of interface controls is provided in figure 2.

### 3.3. HFO detection

**Figure 3.**
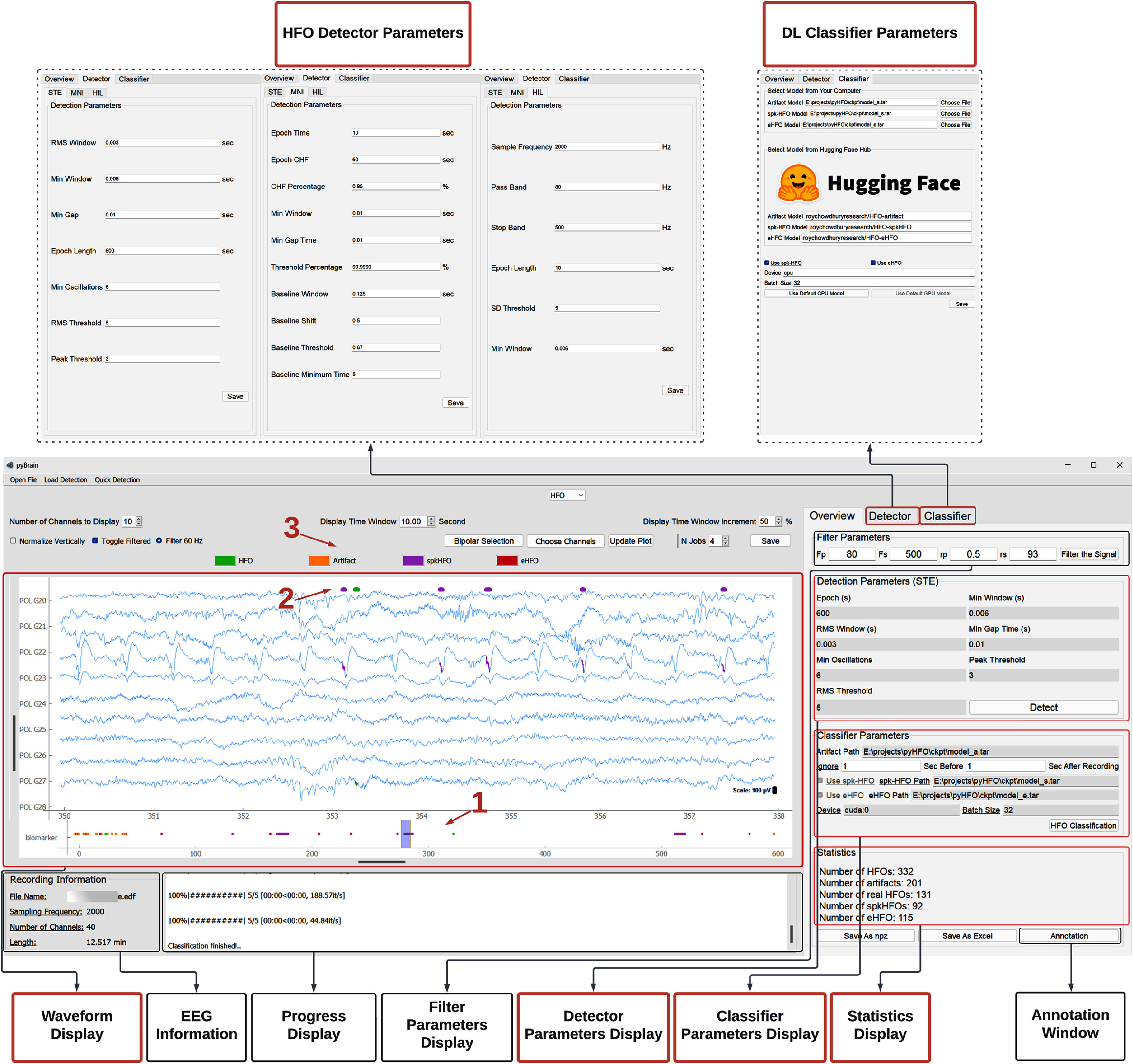
HFO event detection and classification Key features (highlighted with arrows and red boxes) include the visualization of both HFO detection and classification results. Users can configure detector parameters in the ‘Detector’ tab and save them via the ‘Save’ button, with updated settings reflected in the Detector Parameters Display panel. Once detection is performed, users can view the counts for each event category: total detected HFOs, HFOs classified as artifacts, real HFOs (non-artifacts), spkHFOs, and eHFOs. Individual events are overlaid with distinct colors on the signal traces in the waveform display panel (indicated by arrows 1–3). Below the waveform, the event display provides a global timeline of all detections across the recording, enabling rapid navigation to events of interest. Together, these features provide users with a clear and intuitive visualization of the HFO events.

PyHFO 2.0 integrates three established detectors: short-term energy (STE), Montreal Neurological Institute (MNI), and Hilbert, each with user-configurable parameters. Users can select their preferred detector and specify parameter values. Once applied, detected events are automatically marked on the waveform display, with summary statistics reported in the ‘Statistics Display’ panel. An event timeline view further facilitates navigation across detections, helping users quickly locate events of interest. These features allow a balance between automated detection and transparent visualization. A detailed breakdown of interface controls is provided in figure 3.

### 3.4. Biomarker classification

PyHFO 2.0 provides a DL-based HFO classification function, utilizing advanced models to ensure high accuracy and reliability. We adopted the same artifact and spkHFO classifier design as presented in a previous study, along with the eHFO classifier, given their strong performance compared to expert annotations. To ensure seamless integration and accessibility, we have uploaded our model checkpoint to the Hugging Face platform, allowing users to easily download the pre-trained model directly. PyHFO 2.0 leverages the Hugging Face API to run the inference process, streamlining the user experience. This integration simplifies the setup process and enables users to take advantage of state-of-the-art models with minimal effort. In addition, if users wish to train and deploy their own classification models in the future, the process remains standardized. By adhering to Hugging Face’s framework, users can upload their custom-trained models to the platform and integrate them into PyHFO 2.0 following the same procedure.

**Figure 4.**
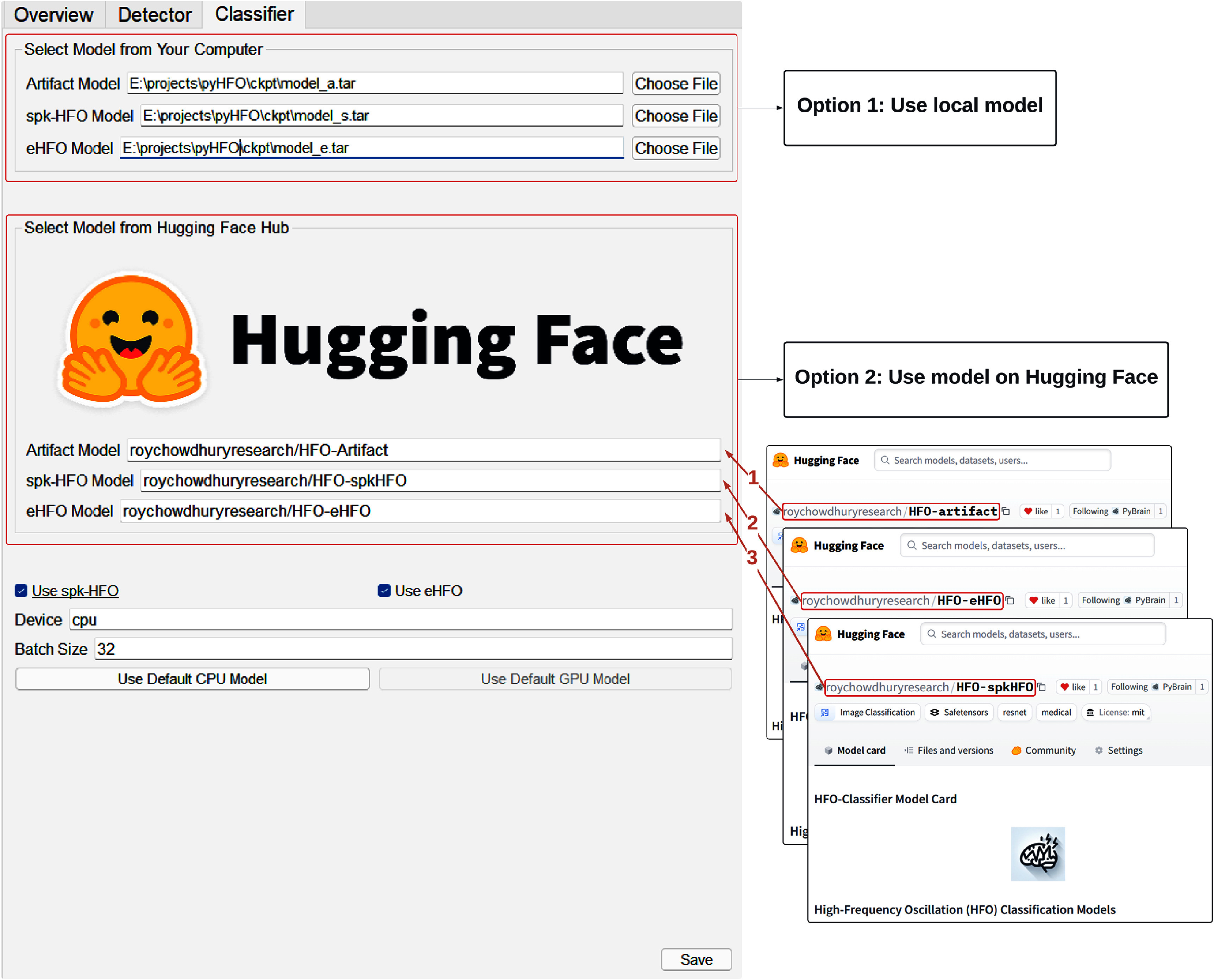
Deep learning classifier parameters setup window. Users can specify the model path using one of two options: (1) select an existing model from the local machine, or (2) provide the model card name from the Hugging Face Hub (arrow 1–3). If users do not wish to use their own models, they can simply click on the ‘Use Default CPU Model’ button or the ‘Use Default GPU Model’ button (if a GPU is available), and all default parameters will be automatically loaded.

To use the classifier, users can navigate to the ‘Classifier’ tab and choose between two options for model loading: Option 1, specify the model path stored locally on the machine; or Option 2, specify the name of the model card stored on Hugging Face (see arrows 1–3 in figure 4). Users can then choose whether to run the model on a CPU or GPU (if available) and set the batch size. For convenience, two buttons, ‘Use Default CPU Model’ and ‘Use Default GPU Model,’ are provided for quick parameter configuration. After clicking the ‘Save’ button, the selected parameters are displayed in the ‘Classifier Parameter Display’ panel.

When the ‘HFO Classification’ button is clicked, if the user selects Option 2 for model loading, the pre-trained model is automatically downloaded (stored in the local Hugging Face cache directory by default), and the classifier begins its inference process. The results, including the number of artifacts, the number of spikes, and the number of real HFOs, are displayed in the ‘Statistics Display’ panel. Additionally, each detected HFO in the waveform panel is updated with a color code corresponding to its classified type: orange for artifacts, purple for spkHFOs, and green for HFOs, as shown in figure 3.

### 3.5. Result export and import

Prediction results can be saved in npz (NumPy) file format or Excel format using the ‘Save As npz’ or ‘save as excel’ options, respectively. Previous run results can also be imported via the ‘Load Detection’ button, allowing users to select a previously saved npz or Excel file.

### 3.6. Event annotation

**Figure 5.**
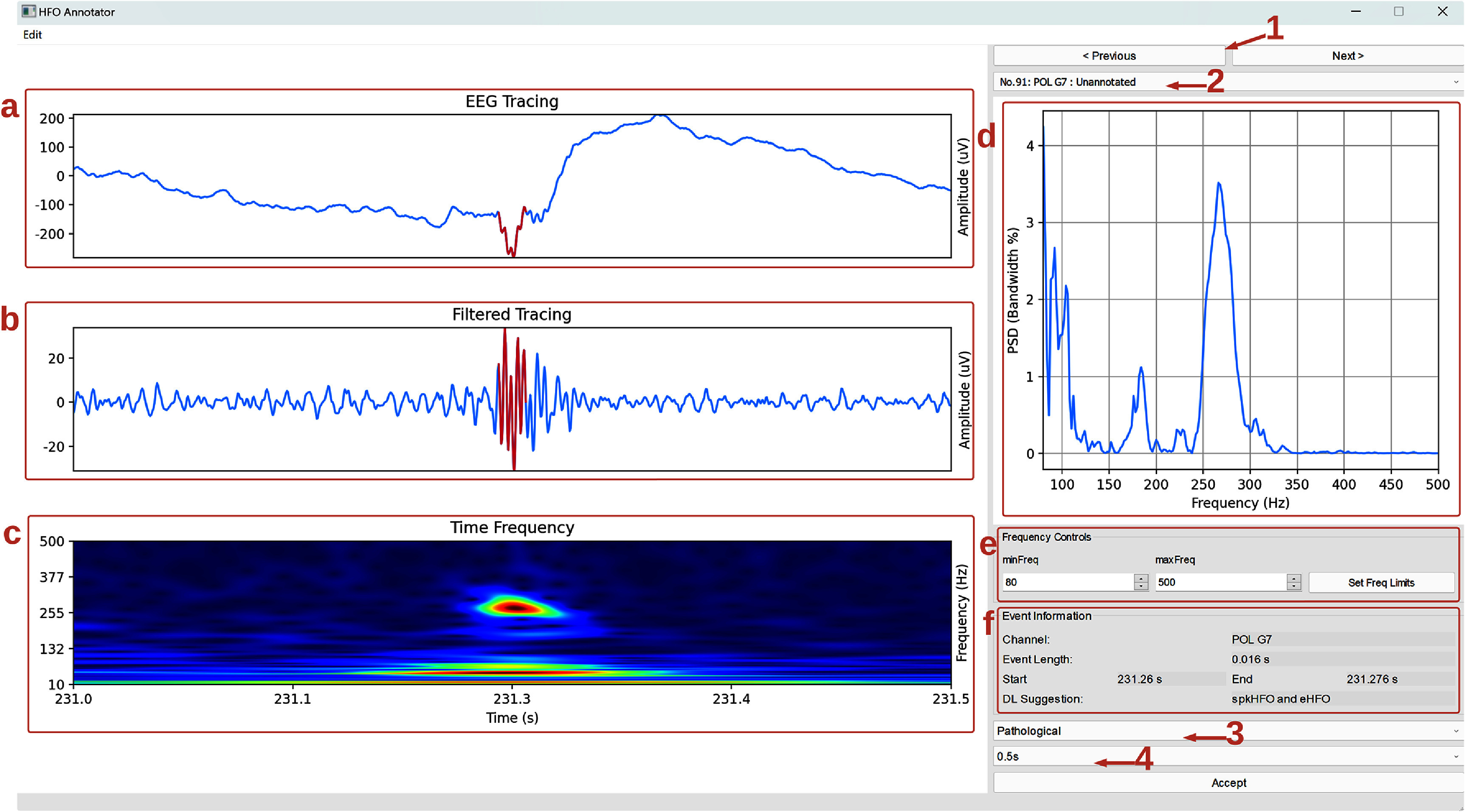
Annotation window. Screenshot of the Annotation interface in PyHFO 2.0. The raw signal, filtered signal, time–frequency plot, and FFT plot (highlighted boxes (a)–(d)) show a signal segment centered on the midpoint of the detected HFO. The ‘Duration Selection’ control (arrow 4) and the ‘Frequency Controls’ (highlighted box (e) enable users to refine the displayed time and frequency ranges for a more detailed view of each event. Arrows 1 and 2 indicate the ‘Previous’, ‘Next’, and ‘Event Selection’ controls for navigating among HFO events. Arrow 3 points to the ‘Event Type’ drop-down menu, which allows users to update annotations. Additionally, box e includes the ‘Event Information’ panel, displaying details such as the HFO’s channel, duration, start and end times, and classifier-predicted type.

PyHFO 2.0 also provides an annotation window, which can be accessed by clicking the ‘Annotation’ button. This feature opens in a pop-up interface that allows users to review each detected HFO in detail. For every event, the interface displays four signal views: the raw signal, the filtered signal, a time–frequency plot (with a user-defined frequency range), and an FFT plot (also with a user-defined frequency range), all centered on the midpoint of the HFO duration (see highlighted boxes (a)–(d) figure 5). Both time and frequency parameters can be adjusted for more precise visualization. The ‘Duration Selection’ drop-down menu (arrow 4 in figure 5) allows users to set the signal window to 1 s, 0.5 s or 0.25 s. The ‘Frequency Controls’ section (highlighted box e in figure 5) lets users modify the frequency display range for both the time–frequency and FFT plots. In the ‘Event Information’ panel (highlight box e in figure 5), users can view key details about the current HFO, including its channel, duration, start and end times, and the classifier predicted type (DL Suggestion). To annotate the event, users may select a new event type from the ‘Event Type’ drop-down menu (arrow 3 in figure 5) and click the ‘Accept’ button. Navigation between detected HFOs is facilitated by the ‘Previous’ and ‘Next’ buttons (arrow 1 in figure 5) and the ‘Event Selection’ drop-down menu (arrow 2 in figure 5).

### 3.7. Quick detection

**Figure 6.**
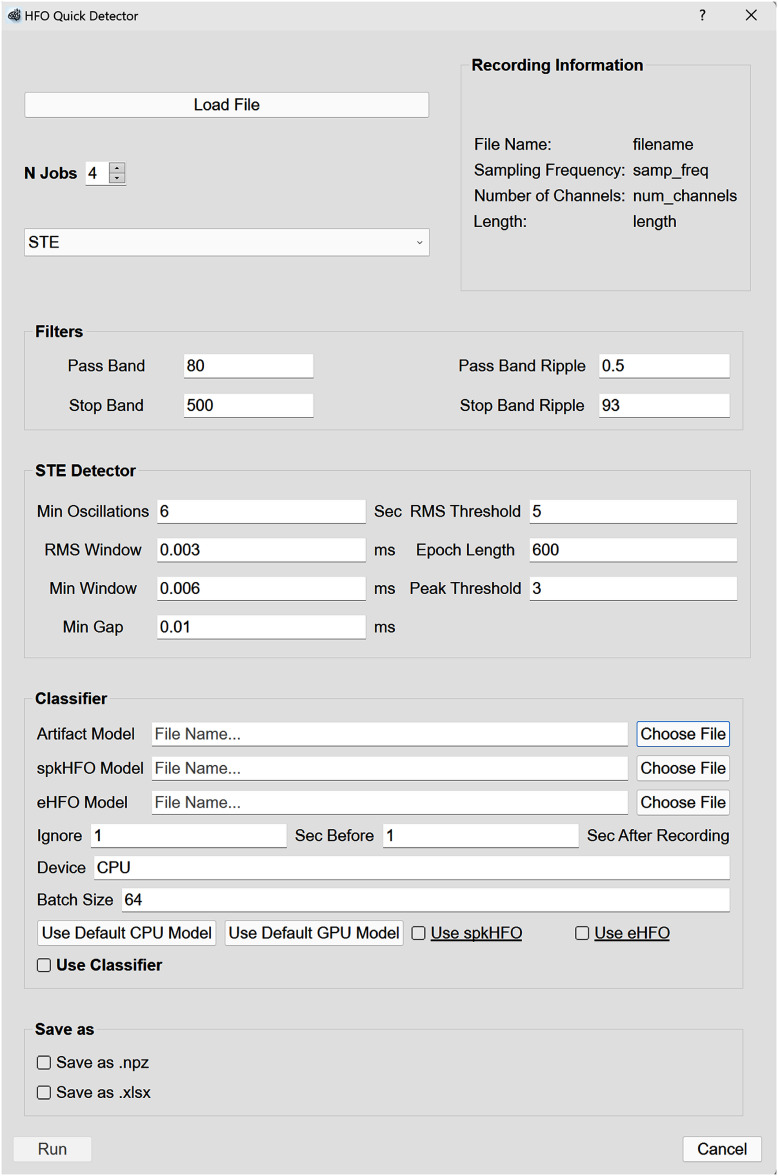
Quick detector window. This streamlined interface allows users to configure the entire detection and classification workflow within a single pop-up window. Users can select an EDF file, adjust filtering parameters, choose a detection method, and optionally enable classification and result export. Once all settings are defined, clicking the ‘Run’ button sequentially executes the chosen modules, generating the final results.

PyHFO 2.0 offers a streamlined workflow that allows users to progress from loading an EDF file to exporting HFO results within a single interface. By clicking the ‘Quick Detection’ button, users can configure all necessary settings through a single pop-up window (figure 6). The process consists of the following steps:

• Select the EDF file.
• Configure filter parameters.
• Choose a detection method and set detector parameters.
• (Optional) enable classification, select model checkpoints, and configure classification parameters.
• (Optional) enable result export and select the desired file format.

Once all parameters are set, clicking the ‘Run’ button sequentially executes the chosen modules, processing the EEG data, and generating the final results.

### 3.8. Deploying DL models

PyHFO 2.0 includes three built-in DL models for artifact detection, spkHFO classification, and eHFO classification. These pretrained models provide strong baseline performance, but like all machine learning approaches, their generalization is influenced by the characteristics of the datasets on which they were trained. For underrepresented or domain-specific EEG data, users may need to fine-tune the models or retrain them to achieve optimal performance. To promote this flexibility and research extensibility, the platform enables users to replace these models with their own. This section outlines the standardized steps for training and integrating compatible models and demonstrates the integration process using an existing eHFO classifier.

#### 3.8.1. Model training requirements and input format

Users are free to define their own model architecture and training datasets, but to ensure compatibility, the model input format must follow the standardized feature extraction process implemented in the software. Each detected HFO event is represented by a time–frequency plot derived from a two-second EEG segment centered at the midpoint of the event. A wavelet transform is applied to obtain the time–frequency representation, covering a frequency range of 10–500 Hz sampled at 224 frequency points. To focus on the event itself, the outmost 0.5 s before and 0.5 s after the HFO event are removed from the original two-second segment, leaving a final time–frequency representation spanning 224 frequency bins across a one-second duration, with the HFO event centered. This representation is then resized to a 224 × 224 image, which serves as the input of the model. Users must ensure that their training data aligns with this format to integrate seamlessly into the classification pipeline.

#### 3.8.2. Model integration pathways

Once a model is trained, it can be integrated through two methods. The first option is local integration, where the trained model is saved in a PyTorch compatible format, such as .pt or .pth, and placed in the ckpt folder within the software directory. The second option is the integration of the Hugging Face Hub, which provides a cloud-based model hosting approach. Users can either train a model using the Hugging Face API, for which a sample training script (train_example.py) is provided on GitHub, or migrate an existing trained model by defining a Hugging Face model class and transferring the model weights. A migration script (migrate_example.py) is available to assist in this process.

By following these steps, users can replace any of the default classifiers with their own models, enabling a flexible and extensible classification framework. In our implementation, the eHFO model was migrated using migrate_example.py while the artifact and spkHFO models were updated with new data using the train_example.py script.

#### 3.8.3. Demonstration: eHFO model distillation and deployment

To demonstrate how an existing DL model can be integrated into the PyHFO 2.0 framework, we selected the publicly available eHFO classifier as a case study. In the original implementation, a 1000 ms EEG segment centered around the HFO event midpoint was extracted and transformed into three distinct image representations: a time–frequency plot (via Gabor wavelets from 10 to 500 Hz), a tracing plot (time-domain waveform mapped onto a 2000 × 2000 image), and an amplitude-coding plot (where pixel intensity reflected signal amplitude over time). These three images were resized to 224 × 224 and concatenated to form a multi-channel input for classification.

To align this model with our standardized pipeline and reduce computational overhead, we performed a model distillation. We retained the original architecture but limited the input to a single time–frequency plot generated under the same frequency and time window (10–500 Hz over 1000 ms). This time–frequency representation was duplicated across the channel dimension to yield a 3×224×224 input, ensuring compatibility with the expected input shape of the original model.

We used the Open-iEEG dataset [36], which includes recordings from 185 epilepsy patients and 686 410 HFO events detected by the STE and MNI detectors, for distillation. After removing artifacts using the PyHFO artifact classification model, 332 409 valid events remained. Predictions from the original eHFO model were used as pseudo-labels to train the distilled version. This approach maintained high predictive accuracy while eliminating reliance on multiple image modalities and reducing computational cost.

Since the distilled eHFO model relies solely on the time–frequency plot input, it can be seamlessly integrated into the PyHFO platform by incorporating the relevant model definition and loading the corresponding checkpoints. The remaining procedures align with those of existing classification models, showcasing the ease of introducing new classification methods.

### 3.9. Verification of the HFO detector

To verify the newly integrated Hilbert detector in PyHFO 2.0, we followed the same validation procedure as in PyHFO by comparing the detection results with those of RIPPLELAB on three datasets (UCLA, Zurich, and Rodent), using the parameters listed in table 2. Table 1 presents the number of HFOs detected by all PyHFO 2.0 detectors: STE, MNI, and Hilbert in two experimental settings. In the ‘Hybrid’ setting, RIPPLELAB performed data reading and bandpass filtering, while PyHFO detection was handled by PyHFO 2.0. In the ‘PyHFO 2.0’ setting, all data reading, bandpass filtering, and detection were performed exclusively by PyHFO 2.0. Events are deemed exactly the same if they overlap by 100%, while rows 90% overlap and 50% overlap indicate how many events align for at least 90% or 50% of their intervals, respectively. Overall, these findings confirm that the Hilbert detector in PyHFO 2.0 closely aligns with RIPPLELAB, mirroring the strong agreement also observed for the previously implemented STE and MNI detectors.

**Table 1.** Event comparison of differences between RIPPLELAB and PyHFO 2.0 implementations in UCLA, Zurich, and Rodent datasets. **Hybrid:** Data reading and bandpass filtering were performed using RIPPLELAB; detection was performed using PyHFO 2.0. **PyHFO 2.0:** All data reading, bandpass filtering, and detection were performed using PyHFO 2.0. **new-RIPPLELAB** and **new-PyHFO 2.0** indicate events detected only by the respective detector ($\unicode{x2A7E} 50\%$ overlap criterion). The difference between 90% and 50% overlap $\left( \frac{n_{{50\%}} - n_{{90\%}}}{n_{{90\%}}} \right)$ is minimal ($\unicode{x2A7D} 0.2\%$).

	No. Events From support detectors								
	STE ddetector			MNI detector			Hilbert detector		
	RIPPLELAB	Hybrid	PyHFO 2.0	RIPPLELAB	Hybrid	PyHFO 2.0	RIPPLELAB	Hybrid	PyHFO 2.0
UCLA									

Total HFO	12 494	12 494	12 501	10 392	10 390	10 355	38 206	38 206	37 955
Exactly Same	—	12 494	8643	—	10 368	7487	—	38 206	18 749
90% overlap	—	12 494	11 876	—	10 390	9612	—	38 206	35 933
50% overlap	—	12 494	11 892	—	10 390	9619	—	38 206	35 975
new-RIPPLELAB	—	0	602	—	2	773	—	0	2230
new-PyHFO 2.0	—	0	609	—	0	736	—	0	1979

Zurich									

Total HFO	31 744	31 744	31 869	70 988	70 538	71 067	38 135	38 135	38 021
Exactly Same	—	31 744	20 656	—	70 496	50 939	—	38 135	20 833
90% overlap	—	31 744	29 775	—	70 532	65 739	—	38 135	36 185
50% overlap	—	31 744	29 811	—	70 532	65 834	—	38 135	36 212
new-RIPPLELAB	—	0	1933	—	456	5154	—	0	1921
new-PyHFO 2.0	—	0	2059	—	6	5233	—	0	1807

Rodent									

Total HFO	375	375	378	42	42	39	50	50	47
Exactly Same	—	375	325	—	42	31	—	50	35
90% overlap	—	375	370	—	42	39	—	50	47
50% overlap	—	375	370	—	42	39	—	50	47
new-RIPPLELAB	—	0	5	—	0	3	—	0	3
new-PyHFO 2.0	—	0	8	—	0	0	—	0	0

**Table 2.** Detailed parameters of the Hilbert-based HFO detector used for the UCLA, Zurich, and Rodent datasets. filter_freq: Bandpass frequency range (Hz); sd_thres: Detection threshold in standard deviations; min_window: Minimum event duration (s); epoch_len: Epoch length for processing (s).

	UCLA	Zurich	Rodent
filter_freq (Hz)	[80, 500]	[80, 500]	[80, 500]
sd_thres (SD)	5	5	5
min_window (s)	$10 \times 10^{-3}$	$10 \times 10^{-3}$	$10 \times 10^{-3}$
epoch_len (s)	3600	3600	3600

### 3.10. Runtime performance

To assess the computational efficiency of PyHFO 2.0, we benchmarked both the detection and classification modules on the datasets described in section 2.1. All tests were performed on a workstation equipped with an AMD Ryzen 9 9950X3D 16-core processor (4.3 GHz), 64 GB RAM, an NVIDIA GeForce RTX 4090 GPU (32 GB), running Windows 11.

#### 3.10.1. Detection runtime

Table 3 presents a runtime comparison between PyHFO 2.0 and the MATLAB-based counterpart RIPPLELAB on the UCLA, Zurich, and Rodent datasets. Because these datasets differ in the number of recordings, channel counts, and recording durations, we normalized the runtime to a common measure of detection speed: the number of milliseconds required to process one minute of EEG data from a single channel (120k samples at a 2000 Hz sampling rate). Using identical detection parameters, PyHFO 2.0 significantly outperformed RIPPLELAB in both single-core (*n* = 1) and multi-core (*n* > 1) configurations. The runtime per channel minute is not identical across datasets because certain preparation steps, such as opening files and initializing filters, take a similar time regardless of recording length. In smaller recordings, these fixed steps form a larger fraction of the runtime, whereas in larger recordings they are spread over more data. Therefore, the values in table 3 should be viewed as comparative benchmarks rather than exact predictors for all dataset sizes.

**Table 3.** Comparative analysis of runtime (measured in milliseconds per channel-minute) in RIPPLELAB and PyHFO 2.0 on the UCLA, Zurich, and Rodent datasets. The roughly total detection time is shown in parentheses. When *n* > 1, PyHFO runs in a multi-core setup (*n*-jobs = 8 on a Windows machine). The best performance (i.e. the shortest runtime) in each dataset is highlighted in bold. Abbreviations: d: day(s), h: hour(s), m: minute(s), s: second(s).

	Runtime for support detectors		
	STE detector	MNI detector	Hilbert detector
UCLA (40 minute recordings, 1715 channels)			

RIPPLELAB	20.08 (≈3.8 h)	108.71 (≈20.7 h)	19.38 (≈3.7 h)
PyHFO 2.0($n = $1)	1.83 (≈20.9 m)	35.39 (≈6.7 h)	1.92 (≈21.9 m)
PyHFO 2.0($n > $1)	**0.42 (≈4.8 m)**	**8.49 (≈1.6 h)**	**0.47 (≈5.3 m)**

Zurich (1915 minute recordings, 9360 channels)			

RIPPLELAB	1.74 (≈8.7 h)	9.61 (≈2d)	1.85 (≈9.2 h)
PyHFO 2.0($n = $1)	0.18 (≈52.7 m)	2.88 (≈14.4 h)	0.17 (≈51.7 m)
PyHFO 2.0($n > $1)	**0.06 (≈17.7 m)**	**0.81 (≈4.0 h)**	**0.07 (≈19.7 m)**

Rodent (20 minute recordings, 19 channels)			

RIPPLELAB	315.79 (≈2.0 m)	3394.73 (≈21.5 m)	300.00 (≈1.9 m)
PyHFO 2.0($n = $1)	33.42 (≈12.7 s)	904.68 (≈5.7 m)	38.16 (≈14.5 s)
PyHFO 2.0($n > $1)	**15.37 (≈5.8 s)**	**269.63 (≈1.7 m)**	**18.68 (≈7.1 s)**

#### 3.10.2. Classification runtime

Table 4 summarizes the runtime performance of the three built-in classifiers (artifact, spkHFO, and eHFO) in PyHFO 2.0. Inference is event-based: each detected HFO candidate is classified independently. As a result, the per-event inference time is almost constant, and total runtime scales linearly with the number of detected events, enabling straightforward extrapolation to larger datasets. On CPU, classification required on average 0.30–0.34 ms per event for the artifact and spkHFO models and 2.3 ms per event for the eHFO model. GPU inference further reduced runtime to 0.14–0.19 ms per event for the artifact and spkHFO models and 1.59 ms per event for the eHFO model. These results demonstrate that all three classifiers can process large batches of HFO candidates efficiently, with GPU acceleration providing an additional speed-up.

**Table 4.** Runtime performance of classifiers in PyHFO 2.0. Reported values correspond to the average inference time per event (ms/event) for artifact, spkHFO, and eHFO models on CPU and GPU.

	Artifact model	spkHFO model	eHFO model
CPU (ms/event)	0.303	0.337	2.291
GPU (ms/event)	0.138	0.190	1.586

To illustrate how these runtime metrics translate into practice, consider a 5 min EEG recording with 100 channels (500 channel-minutes total). Using PyHFO 2.0 with multi-core processing (*n* = 8, Windows system), the STE and Hilbert detectors require approximately 15–19 ms per channel-minute (table 3), corresponding to about 7–9 s for the full recording, while the more computationally intensive MNI detector completes in ∼135 s. Once candidate HFOs are detected, classification proceeds on an event-by-event basis. With GPU acceleration, inference requires ∼0.14–1.6 ms per event depending on the model (table 4), meaning that 1000 detected candidates can be processed in under 2 s. These results demonstrate that PyHFO 2.0 achieves practical runtimes even on multi-channel, multi-minute EEG recordings.

### 3.11. Clinical validation of DL models

#### 3.11.1. eHFo model deployment evaluation

We evaluated the performance of the distilled eHFO model by comparing it against the original eHFO model on the UCLA dataset. The goal of this evaluation is to verify that the distillation process preserves the predictive performance of the original model while significantly reducing computational overhead. Specifically, we treated the predictions of the original eHFO model as pseudo-labels and assessed how well the distilled model replicates these predictions. Accuracy was used as the evaluation metric. Our results show that the distilled eHFO model closely aligns with the original model, achieving an accuracy of 0.86.

In addition, following the methodology of [31], we computed the computational complexity of both models. The original eHFO model requires 1.822 G MACs, while the distilled version requires only 486.95 M MACs, representing a 73% reduction in computational cost due to distillation.

#### 3.11.2. Model generalization on Hilbert-based HFO detection

To evaluate how well the artifact and spkHFO classifiers generalize to events detected by the Hilbert method, we compared model predictions with expert annotations from five UCLA patients. For this analysis, we directly used the artifact and spkHFO models developed in PyHFO 1.0 without retraining, to test their ability to generalize to detections from the newly added Hilbert method. Each detected event was independently labeled by board-certified epileptologists (AD, SK) into one of three categories: artifact, real HFO without spike, or spkHFO. Specifically, the annotated dataset includes 175 artifact events, 1366 real HFOs, and 2025 spkHFOs. The performance of the model was assessed against these labels. The resulting performance metrics are summarized in table 5. Unlike the STE and MNI detectors, which were evaluated using five-fold cross-validation on the UCLA dataset and therefore report mean values with 95% confidence intervals, the Hilbert results are reported as point estimates since they reflect validation on a separate expert-annotated set. As shown, both classifiers maintained high accuracy, precision, recall, and *F*1 scores.

**Table 5.** Performance analysis using five-fold cross-validation: mean accuracy, *F*1-score, recall, and precision with 95% confidence intervals (CIs) for STE and MNI. For Hilbert, results reflect evaluation on detections from 5 UCLA patients compared against expert annotations; hence, CIs are not reported.

	STE		MNI		Hilbert	
	Artifacts	spkHFO	Artifacts	spkHFO	Artifacts	spkHFO
Accuracy	98.56 ± 0.19	89.18 ± 0.82	98.72 ± 0.64	89.79 ± 3.12	96.41	90.50
*F*1-score	99.08 ± 0.13	91.40 ± 0.60	90.16 ± 4.88	94.14 ± 1.93	98.14	92.14
Recall	99.18 ± 0.27	90.78 ± 1.91	94.79 ± 2.72	90.85 ± 4.15	99.68	93.23
Precision	98.98 ± 0.29	92.07 ± 2.11	100.0 ± 0.00	97.75 ± 0.69	96.65	91.08

#### 3.11.3. Seizure outcome prediction

**Figure 7.**
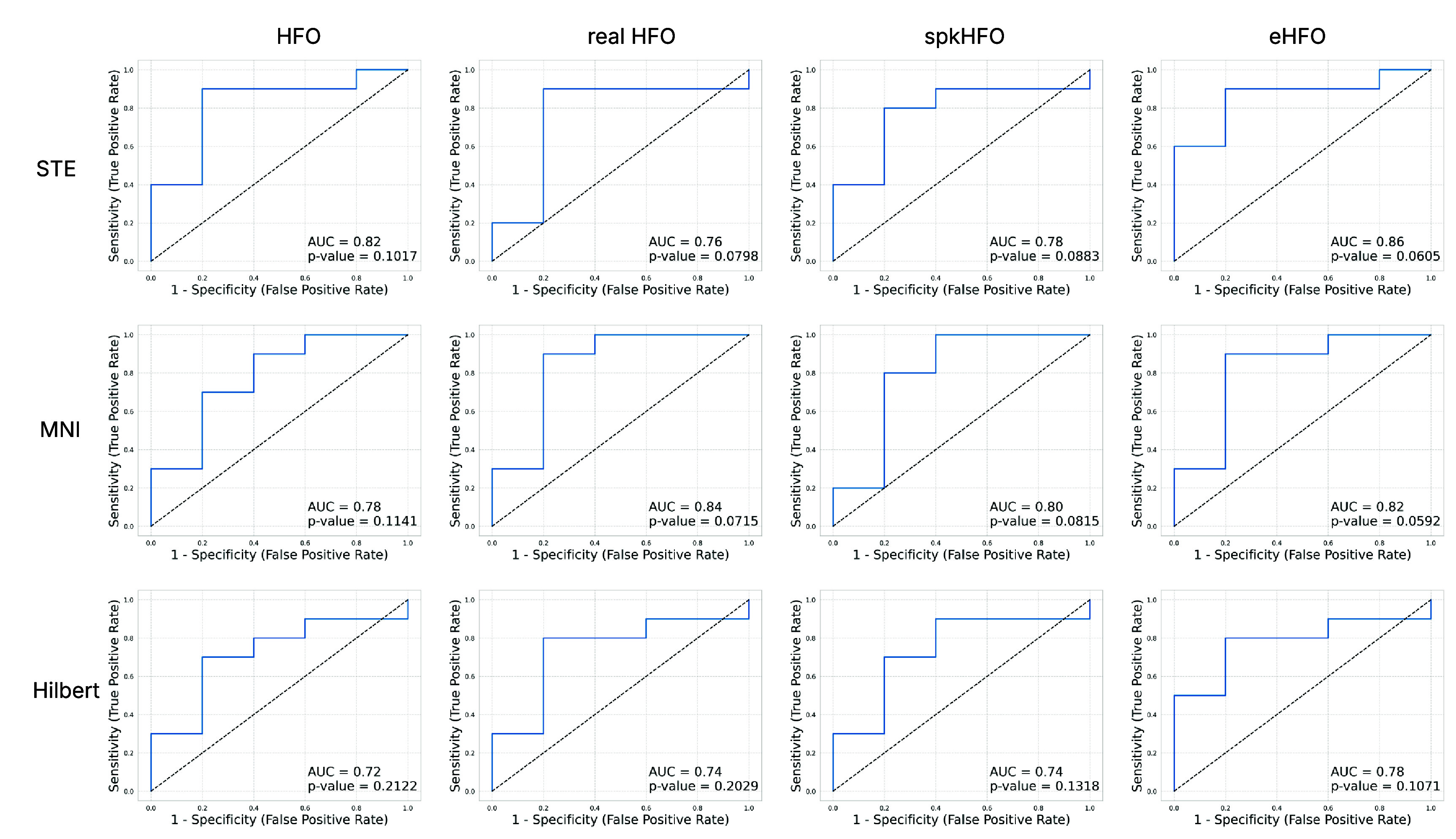
ROC curves. We constructed postoperative seizure outcome prediction models using event resection ratio derived from 10 min EEG data (*n* = 15). Each subfigure illustrates the classification performance for surgical outcomes using area under the ROC curve (AUC) as the evaluation metric, with *p*-values indicating the statistical significance of the relationship between resection ratio and seizure freedom. Rows correspond to events detected by different detectors: STE (top), MNI (middle), and Hilbert (bottom). Columns represent different event types: (1) HFO events, (2) real HFOs identified using the artifact classification model, (3) real spkHFOs identified using both artifact and spike classification models, and (4) real eHFOs identified using the artifact, spike, and eHFO classification models.

Finally, we conducted a clinical evaluation of the three trained detectors and the DL-based HFO classification model by assessing their ability to predict surgical outcomes using the derived biomarkers. Specifically, we used the UCLA dataset as the validation cohort, which includes 15 patients with documented surgical outcomes and corresponding EEG recordings annotated by channel-level resection status. The objective of this evaluation was to build a logistic regression model using the resection ratio as a predictive feature for postoperative seizure outcomes (seizure-free vs. not seizure-free). We report the predictive performance of each biomarker in terms of the area under the ROC curve (AUC), as summarized in (figure 7).

Overall, these results confirm that PyHFO 2.0 combines practical usability with robust detection, efficient DL integration, and clinically relevant biomarkers, motivating the following discussion of its broader implications and limitations.

## 4. Discussion

In this work, we introduced PyHFO 2.0, a user-friendly open-source platform that builds on our original PyHFO framework by integrating modern DL methods and incorporating additional HFO detection algorithms. PyHFO 2.0 streamlines the entire workflow–from EEG loading and filtering to HFO detection, classification, and annotation–through an intuitive graphical interface. By offering the Hilbert detection method, a dedicated eHFO classification model, and seamless integration with the Hugging Face ecosystem, PyHFO 2.0 simplifies the use of advanced machine learning models for both researchers and clinicians. The annotation window further enhances usability by allowing interactive review and refinement of detection results, thereby improving the overall reliability of HFO analysis. In addition to methodological advances, PyHFO 2.0 was designed with an emphasis on practical usability. To support adoption by both clinicians and researchers, we provide detailed descriptions of the interface and workflows, enabling the paper to serve not only as a methodological contribution but also as a practical reference for end-users. While PyHFO 2.0 significantly improves upon the functionality and accessibility of its predecessor, several limitations must be acknowledged:

### 4.1. Data format compatibility

PyHFO 2.0 currently supports reading EEG data only in the EDF and BrainVision format. This limitation may hinder adoption where other standard EEG file types (e.g. BioSemi’s.bdf) are more prevalent, requiring users to convert data before analysis.

### 4.2. Dependence on training data

The performance of the DL-based classification models relies heavily on the representativeness and quality of the training data. Users with highly specialized or underrepresented EEG datasets may need to fine-tune or retrain these models to achieve optimal performance. Future efforts could involve expanding the model’s training datasets or incorporating transfer learning techniques to better handle diverse clinical and research use cases.

### 4.3. Focus on HFOs

Although PyHFO 2.0 was developed primarily for HFO detection and classification, the platform could be extended to identify additional neurophysiological biomarkers. Implementing modules for other events, such as epileptiform discharges, epileptic spasms [37], or sleep spindles, broadens its applicability and potentially benefits a wider range of studies in clinical neurophysiology.

### 4.4. Image-based representation of HFOs

The current DL classification pipeline assumes that biomarkers to be classified can be represented in an image-like format (for example, through time–frequency spectrograms). While this approach works well for many HFO-related events, it may be less suitable for biomarkers that do not neatly lend themselves to two-dimensional representations. Additional preprocessing steps or alternative pipeline designs may be necessary to support a broader range of biomarker signals.

### 4.5. Offline-only processing

PyHFO 2.0 is currently limited to offline analysis and does not support streaming or real-time data processing. This restricts potential applications such as intraoperative monitoring and closed-loop neuromodulation.

### 4.6. Future directions

Moving forward, we plan to enhance the waveform display in both the main GUI and the annotation window by introducing interactive cursors, zooming, and improved navigation tools, making it easier to inspect specific signal segments. We also intend to expand the platform to detect and classify additional neurophysiological biomarkers, with spindle detection as a priority target for future implementation. In addition, integrating newer DL techniques, such as transfer learning approaches, could improve classification performance across various data sources. Finally, an important direction is real-time processing. Platforms such as OpenViBE [38] and the FieldTrip real-time module [39] have demonstrated the feasibility of online EEG streaming and analysis. Given the fast detection and classification runtimes achieved in PyHFO 2.0, extending the framework to real-time operation is technically feasible and could broaden its clinical utility, for example in intraoperative monitoring or closed-loop neuromodulation.

Overall, PyHFO 2.0 represents a significant advancement in bridging the gap between DL innovations and clinical neuroscience. By providing a flexible, intuitive, and open-source framework for HFO detection, classification, and annotation, PyHFO 2.0 equips researchers and clinicians with a powerful tool to accelerate and enrich biomarker analysis. To ensure long-term viability, the platform will be actively maintained as an open-source project on GitHub, with regular updates guided by user feedback and contributions from the community.

## Data Availability

The data that support the findings of this study are openly available at the following URL/DOI: https://openneuro.org/datasets/ds003498/versions/1.1.1; https://github.com/roychowdhuryresearch/pyHFO/tree/pyBrain.

## References

[1] Weiss S A (2018). Visually validated semi-automatic high-frequency oscillation detection aides the delineation of epileptogenic regions during intra-operative electrocorticography. Clin. Neurophysiol..

[2] Boran E, Ramantani G, Krayenbühl N, Schreiber M, König K, Fedele T, Sarnthein J (2019). High-density ECOG improves the detection of high frequency oscillations that predict seizure outcome. Clin. Neurophysiol..

[3] Dimakopoulos V, Mégevand P, Boran E, Momjian S, Seeck M, Vulliémoz S, Sarnthein J (2021). Blinded study: prospectively defined high-frequency oscillations predict seizure outcome in individual patients. Brain Commun..

[4] Zweiphenning W (2022). Intraoperative electrocorticography using high-frequency oscillations or spikes to tailor epilepsy surgery in the Netherlands (the HFO trial): a randomised, single-blind, adaptive non-inferiority trial. Lancet Neurol..

[5] Jacobs J (2018). Removing high-frequency oscillations: a prospective multicenter study on seizure outcome. Neurology.

[6] Akiyama T (2011). Focal resection of fast ripples on extraoperative intracranial EEG improves seizure outcome in pediatric epilepsy. Epilepsia.

[7] Wu J, Sankar R, Lerner J, Matsumoto J, Vinters H, Mathern G (2010). Removing interictal fast ripples on electrocorticography linked with seizure freedom in children. Neurology.

[8] Jacobs J, Zijlmans M, Zelmann R, Chatillon C-E, Hall J, Olivier A, Dubeau F, Gotman J (2010). High-frequency electroencephalographic oscillations correlate with outcome of epilepsy surgery. Ann. Neurol..

[9] van’t Klooster M A (2017). Tailoring epilepsy surgery with fast ripples in the intraoperative electrocorticogram. Ann. Neurol..

[10] Monsoor T (2023). Optimizing detection and deep learning-based classification of pathological high-frequency oscillations in epilepsy. Clin. Neurophysiol..

[11] Zhang Y (2022). Characterizing physiological high-frequency oscillations using deep learning. J. Neural Eng..

[12] Zhang Y (2022). Refining epileptogenic high-frequency oscillations using deep learning: a reverse engineering approach. Brain Commun..

[13] Foffani G, Uzcategui Y G, Gal B, de la Prida L M (2007). Reduced spike-timing reliability correlates with the emergence of fast ripples in the rat epileptic hippocampus. Neuron.

[14] Li Z (2025). Concordant interictal stereoelectroencephalographic high-frequency oscillations and magnetoencephalography predict better surgical outcomes in focal epilepsy. Epilepsia.

[15] Li X (2025). Single-neuron discharges correlating high-frequency oscillations dynamics in epileptogenesis and epilepsy development. J. Neurosci. Res..

[16] Zhang Y, Ding Y, Duan C, Daida A, Nariai H, Roychowdhury V (2025). Self-supervised distillation of legacy rule-based methods for enhanced EEG-based decision-making. https://arxiv.org/abs/2507.14542.

[17] Daida A, Zhang Y, Kanai S, Staba R, Roychowdhury V, Nariai H (2025). AI-based localization of the epileptogenic zone using intracranial EEG. Epilepsia Open.

[18] Mishra A (2025). Motifs of human high-frequency oscillations structure processing and memory of continuous audiovisual narratives. Sci. Adv..

[19] Karatza P, Cserpan D, Moser K, Lo Biundo S P, Sarnthein J, Ramantani G (2025). Scalp high-frequency oscillation spatial distribution is consistent over consecutive nights, while rates vary with antiseizure medication changes. Epilepsia.

[20] Ryvlin P (2025). SEEG in 2025: progress and pending challenges in stereotaxy methods, biomarkers and radiofrequency thermocoagulation. Curr. Opin. Neurol..

[21] Navarrete M, Alvarado-Rojas C, Le Van Quyen M, Valderrama M, Charpier S (2016). RIPPLELAB: a comprehensive application for the detection, analysis and classification of high frequency oscillations in electroencephalographic signals. PLoS One.

[22] Nariai H (2020). Scalp EEG interictal high frequency oscillations as an objective biomarker of infantile spasms. Clin. Neurophysiol..

[23] Gliske S V (2018). Variability in the location of high frequency oscillations during prolonged intracranial EEG recordings. Nat. Commun..

[24] Kuroda N, Sonoda M, Miyakoshi M, Nariai H, Jeong J-W, Motoi H, Luat A F, Sood S, Asano E (2021). Objective interictal electrophysiology biomarkers optimize prediction of epilepsy surgery outcome. Brain Commun..

[25] Lisgaras C P, Scharfman H E (2023). Epilepsia.

[26] Barth K J (2023). Flexible, high-resolution cortical arrays with large coverage capture microscale high-frequency oscillations in patients with epilepsy. Epilepsia.

[27] Petito G (2022). Diurnal rhythms of spontaneous intracranial high-frequency oscillations. Seizure.

[28] Gramfort A, Hämäläinen M S (2013). MEG and EEG data analysis with MNE-Python. Front. Neurosci..

[29] Vallat R, Walker M P (2021). An open-source, high-performance tool for automated sleep staging. eLife.

[30] Bao F S (2011). PyEEG: an open source python module for EEG/MEG feature extraction. Comput. Intell. Neurosci..

[31] Zhang Y (2024). PyHFO: lightweight deep learning-powered end-to-end high-frequency oscillations analysis application. J. Neural Eng..

[32] Nariai H (2019). Prospective observational study: fast ripple localization delineates the epileptogenic zone. Clin. Neurophysiol..

[33] Fedele T, Burnos S, Boran E, Krayenbühl N, Hilfiker P, Grunwald T, Sarnthein J (2017). Resection of high frequency oscillations predicts seizure outcome in the individual patient. Sci. Rep..

[34] Santana-Gomez C (2019). Harmonization of pipeline for detection of HFOs in a rat model of post-traumatic epilepsy in preclinical multicenter study on post-traumatic epileptogenesis. Epilepsy Res..

[35] Crépon B, Navarro V, Hasboun D, Clemenceau S, Martinerie J, Baulac M, Adam C, Le Van Quyen M (2009). Mapping interictal oscillations greater than 200 Hz recorded with intracranial macroelectrodes in human epilepsy. Brain.

[36] Zhang Y (2025). Self-supervised data-driven approach defines pathological high-frequency oscillations in epilepsy. Epilepsia.

[37] Daida A (2025). Evidence of thalamocortical network activation during epileptic spasms: a thalamic stereotactic EEG study. Epilepsia.

[38] Renard Y, Lotte F, Gibert G, Congedo M, Maby E, Delannoy V, Bertrand O, Lécuyer A (2010). OpenViBE: an open-source software platform to design, test and use brain–computer interfaces in real and virtual environments. Presence: Teleoperators Virtual Environ..

[39] Oostenveld R, Fries P, Maris E, Schoffelen J-M (2011). FieldTrip: open source software for advanced analysis of MEG, EEG and invasive electrophysiological data. Comput. Intell. Neurosci..

